# 

**Published:** 1870-12

**Authors:** 


VOL. XXIV. No. 12.

### THE Dental Register.

EDITED BY

J. TAFT & G. WATT.

DECEMBER, 1870.

MONTHLY:

THREE DOLLARS PER ANNUM, IN ADVANCE.

CINCINNATI:

WRIGHTSON & CO., Printers, 167 WALNUT STREET.

J. & WM. TAFT. Dentists. 117 West Fourth St.

